# Standing Their Ground While Seated: Narratives of Widowhood Activism Against the Ritual of Widow Dispossession in South Africa

**DOI:** 10.1177/00302228241305652

**Published:** 2024-11-29

**Authors:** Thatshisiwe Ndlovu

**Affiliations:** 1 Narrative Enquiry for Social Transformation (NEST), University of the Witwatersrand, Johannesburg, South Africa

**Keywords:** widowhood, ritual, dispossession, activism, narrative, resistance, sitting

## Abstract

Widows in Africa are mostly perceived as helpless victims of harmful widowhood practices. The article offers new insights into the understanding of widowhood and the status of widows in the context of these practices. Integrating feminist research and narrative theory, the paper examines widows’ responses to the ritual of the ‘sitting and its associated practice of widow dispossession. I examine how widows challenge the customary ritual of ‘sitting’ from a place where enforced silences and widow dispossession are ritualized to where widowhood activism is born. The article draws from a feminist theory of activism, highlighting that widows counter silencing and dispossession through speaking out, speaking through objects, delegating someone to act on their behalf, disobedience and non-cooperation. In summary, the article concludes that the practice of ‘sitting’ is a politically charged space that is both a site of widow dispossession and resistance.

## Introduction

In contemporary Africa, widowhood is indisputably gendered (Akinduyo & Theron, 2024; Fasanmi & Ayivor, 2021) and some African widowhood practices can be construed as a form of violence against women (Fasanmi & Ayivor, 2021; Ude & Njoku, 2017). Following the death of a spouse, women are expected to undergo a myriad of mourning practices and rituals (Mabunda & Ross, 2023; Manala, 2015; Motsoeneng & Modise, 2020). Widowhood rites have been extensively documented and analysed (Mabunda & Ross, 2023; Pauw, 1990; Rosenblatt & Nkosi, 2007; Somhlaba & Wait, 2009; Sossou, 2002). There is a general acknowledgement in death studies scholarship that certain widowhood practices in Africa have been reappropriated into harmful cultural traditions (Fasanmi & Ayivor, 2021; Motsoeneng & Modise, 2020). These works predominantly highlight the gender discrimination embedded in the widowhood rites. While sizeable attention has been given to the descriptive accounts of widowhood practices and how they affect women in Africa and South Africa, existing literature does not capture the nuances and dynamics around how the widows engage with their pain and trauma.

The mourning custom of the ‘sitting’ and widow dispossession are some of the practices that have been construed as harmful to women (Dube, 2023; Kotzé et al., 2012) as they often reinforce and institutionalize systemic inequalities that profoundly impact widows’ lives. In many African societies, widow dispossession (the practice of depriving widows of their rights to property, financial resources, and social status after the death of a spouse) is deeply rooted in cultural, social, economic and sometimes legal practices. This practice often leaves widows in financial distress, socially marginalized, and without access to land and property rights (Dube, 2023). The UN highlights that in patriarchal societies, widows often face gender-based discrimination, are deprived of inheritance rights, and remain vulnerable to economic hardship and abuse (UN Women, 2021). These women may face barriers not only in owning property but also in maintaining autonomy and stability due to discriminatory norms and family structures that reinforce dependency or isolation.

In South Africa, legal reforms have aimed to protect women from dispossession and disinheritance as a strategy to combat poverty and gender inequality.^1^ However, the situation for widows remains challenging due to the coexistence of religious and customary inheritance practices within a pluralistic legal system. This complexity suggests that civil law reforms alone may be insufficient to address the issue fully.

The core focus of this research is to examine the multifaceted responses of widows to the cultural practice of the ‘sitting’ and the related custom of widow dispossession. The paper asks, how do widows experience, navigate, and challenge the ritual of the ‘sitting’ and the dispossession that often accompanies this practice? By exploring these, the study provides a deeper, more nuanced understanding of widowhood that challenges the stereotypes of widows as silent victims of harmful practices. This insight not only raises awareness and support for widows but also highlights pathways to dismantling oppressive customs and fostering more inclusive communities. A narrative inquiry into the lived experiences of widowhood may shed light on how widows navigate and encounter these practices, the impact of their actions on social norms and how these resistance efforts can be supported to dismantle unjust customs within South African society. This article examines how the practice of “sitting” leads to the dispossession of widows’ property and diminishes their status within the family. It also highlights how widows assert agency, shedding light on their activist responses and resistance to these oppressive customs. From this perspective, the widowhood practice of the ‘sitting’ is transformed from a space of enforced silence and dispossession into a platform for widowhood activism and empowerment. The contribution of this paper lies in shifting the understanding of widowhood practices from being seen solely as points of deprivation to processes of restitution, adding important dimensions to the rituals surrounding widowhood and the roles of widows.

## The Practice of ‘Sitting’

While post-apartheid South Africa has seen exponential growth in the black capitalist class (Kotzé et al., 2012) which has led to the wealth divide and the introduction of new mourning practices, and COVID-19 altered some of the customs (Kgadima & Leburu, 2024), the custom of the ‘sitting’ has remained constant, especially in the poorer communities. The ‘sitting’ is a common practice/ ritual done after a person’s death and before the funeral. This practice typically requires the widow to sit on a mat or mattress, often facing the wall and wrapped in a blanket (Kotzé et al., 2012, p. 754). Here, she sleeps alongside a few chosen female relatives. The only time she is permitted to leave the sitting position is to use the bathroom, otherwise she must stay seated at all other times (Rosenblatt & Nkosi, 2007, p. 78). During this period, women are expected to express their grief and loss, and importantly, it serves as a time to recount in detail how the spouse passed away. The ‘sitting’ is a political process, masked by custom and regulated by in-laws who must be satisfied with the accounts given. In this process, the in-laws and other interested parties can lay demands on the grieving widow,^2^ depending on their level of satisfaction with the explanations given to them.

While the original meaning of the ‘sitting’ and other widowhood practices was to allow the widow space to grieve and heal (Nwoye, 2005), the meaning of the ‘sitting’, like all other reinterpreted rituals has been reimagined to a more violent practice of mourning. Like other colonized regions, South Africa experienced the profound effects of colonialism and Western imperialism, which permeated politics, production, science, technology, religion, and culture. Indigenous African cultures and traditional knowledge systems were dismissed as superstitious, primitive, irrational, and unscientific, reflecting the pervasive devaluation imposed by colonial powers (Mawere, 2014, p. 26). As such, cultures were diverted and remodeled to suit European cultural values. This had profoundly negative effects on people’s social lives, significantly altering and distorting the way they practised their culture and religions (Redding, 2023). Before these distortions, African widowhood practices were coherent and transformative (Nwoye, 2005). Makgahlela et al. (2021, p. 93) described African bereavement practices as providing “healing, continuity, and balance”. This supports Nwoye’s definition of African grief practices as “the patterned ways invented in traditional communities for the successful healing of the psychological wounds and pain of bereaved persons” (Nwoye, 2005, p. 148) and Mabunda and Ross (2023)’ assertion that some mourning rites are aimed at removing bad luck or misfortune or ‘*senyama/ ibhadi’* that the widow supposedly embodies. Therefore, the custom of ‘sitting’ is one such practice that has evolved with the changes in culture and its norms. It has transformed into a custom where other harmful rituals are enforced. These include seclusion and ‘sharing’ of possessions with family (Kotzé et al., 2012; Ramphele, 1996).

## Theoretical Lenses: Activism and Resistance

The narrative extracts in this paper were analysed using the Feminist Theory of Activism. A feminist theory of activism “opens the category of activism to consider actions and activities that, because of their limited geographic reach, normally are considered too insignificant to count as activism and yet do create progressive change in the lives of women, their families, and their communities” (Martin et al., 2007, p. 79). As such, this theory offers a framework for understanding activism on the peripheries, which is often unpublicized, unrecognized, not theorized, and away from the media spotlight (Martin et al., 2007). It allows for a “recognition of multiplicity of forms of activism and activists” and this “may take emphasizing the contingent and contested nature of such activism” (Jenkins, 2017, p. 1445), and understanding activists’ identities as being “messy, complex and multiple” (Chatterton & Pickerill, 2010, p. 479 cited in Jenkins, 2017). I applied this theory to capture those actions and activities that women engage in to resist and or negotiate widowhood rites that they considered harmful to them. This involved carefully listening to the explanations behind their actions and the significance they attributed to those actions. By doing so, I looked for *“ways of seeing and seeing against the grain of those usual ways”* (Davies et al., 2006, p. 100, the original text contains italics). The example of widows’ resistance to the ritual of dispossession through the practice of the ‘sitting’ provides an opportunity to examine the nuanced and often overlooked areas where activism occurs, intertwined with daily routines and the fabric of family life.

Resistance in this paper is analyzed through Scott’s (1985) theory of everyday forms of resistance, which posits that the concept of the ‘everyday’ is particularly valuable for interpreting women’s resistance activities. This framework helps to understand how such activism can occur even in challenging and isolated circumstances. Everyday forms of resistance are shaped in response to power by subverting and negotiating the power structures. Scott describes everyday resistance as consisting of small-scale, individual actions that often go unnoticed by the public, with minimal coordination, planning, or experience involved, and significantly lower risk. He provides examples of these largely covert actions, such as “passive non-compliance, subtle sabotage, evasion, and deception” (Scott, 1985, pp. 6–7). My utilization of the concept of the everyday aligns more closely with what Askins (2015) refers to as “quiet politics,” emphasizing mundane and embodied activities outlined by Horton and Kraftl (2009) as “implicit activisms” which he says are focused on “small acts, kind words, and not too much fuss”.

My work considers small acts and embodied activities such as disobedience, uncooperativeness, and actions through proxy and speaking through objects as forms of implicit activisms. It shifts away from the tendency to center activism on major events, actions, and prominent leaders and thinkers, instead emphasizing the “complex contingent contexts, temporalities, and causal happenings” (Horton & Kraftl, 2009, p. 16) that produced the responses. It highlights that it is through the constricted space of the ‘sitting’ in which resistance occurs. This space allows for and requires a form of politics that is very subdued, capable of subtly challenging established patterns of control and authority and disrupting patriarchal dynamics within family settings. This article builds upon enduring feminist theories that conceptualize everyday spaces as politically charged, with a specific focus on how resistance is manifested and ingrained within the intimate sphere of family life.

## Method

### Study Design

The empirical research that underpins this paper is qualitative and grounded in feminist narrative methodology. I drew on narrative inquiry as a methodology to explore the experiences of widows in context and over time (Clandinin, 2022). Through the narrative method of life story interviews, widows told stories about their lived experiences of the mourning practice of ‘sitting’ and dispossession in a highly patriarchal society. Life story narratives here are conceptualized from a feminist standpoint to allow marginalized widows to contribute to a body of knowledge anchored in their subjective experiences. It allows women to take back their narrative through the research process, and to create a space where counter-naratives can be shared. Informed by a feminist framework, the study focuses on the “unique experiences, struggles, needs and desires of African women” (Alexander- Floyd & Simien, 2006, p. 67) and it allows for an understanding of the intersections of cultural practices and gender issues in widowhood practices. This study relied on the narrative enquiry of life story narratives to allow for an understanding of the “intersections of social forces and lived possibilities” (Squire et al., 2014, pp. 76–78) and “story makes the implicit explicit, the hidden seen, the unformed formed and the confusing clear” (Atikson, 1998, p. 7). As such, this approach provides widows with an opportunity to discuss their hidden, sometimes embarrassing moments as well as to share their stories of overcoming, rather than solely highlighting their hardships and reinforcing dominant victimhood narratives. Their stories were also seen as a gateway through which they see their worlds, and how they interpret events (Savin-Baden & Niekerk, 2007). Narrative research encourages a collaborative approach where researchers and participants continually negotiate the meaning of stories through validation checks during data collection and analysis (Creswell, 2013).

## Setting for the Study

This research was carried out in a community called Ebony Park, a neighborhood in Midrand, located northeast of Johannesburg, South Africa. It lies within Region A of the City of Johannesburg Metropolitan Municipality, next to Ivory Park. It is a low to low- middle-income township established in 1997 as part of Johannesburg’s post-apartheid metropolitan municipality. Ebony Park was established to address the overflow of people from nearby Tembisa and other urban areas, providing additional housing and infrastructure to accommodate the growing population in the region. It is home to people of diverse ethnic groups.

## Sampling

This article is based on life story interviews collected in 2014 as part of my master’s degree project and follow-up conversations were done in 2023. The original interviews consisted of 10 women who had lost their husbands within the past 10 years, and I went back in 2023 while preparing to write this paper and had a conversation about my work and findings with four of the women who were part of the original participants. This was done to “negotiate meaning” (Achebe, 2020) to have them talk back, confirm, disprove and add to my original findings. The selected four were central to this study because of their experiences of the ritual of dispossession and how they adopted other ways to resist the rites that they felt were harmful to them. The broader sample, which is useful in understanding the experience of widowhood practices, included women who identified themselves as members of the following ethnic and language groups, Zulu, Xhosa, Pedi, Swati, Ndebele, and Shangaan. As shown in Table 1, their ages ranged from 35 to 65 and their economic statuses ranged from poor to lower middle class.^3^ These were important differences among the participants, and the age and ethnic variations added an advantage to the diversity and complexity of their experiences and responses to culture and customs. I lived in the same area as the participants and was well-acquainted with two of them. At the time, they were my neighbors and fellow church members, and they knew I was a university student. This relationship helped me build rapport and earn their trust, making it easier for them to discuss sensitive topics like marriage and loss with someone younger. As such, those two were my first participants, and they then referred and recruited other widows in the area. Most of the participants were fluent in IsiZulu and preferred the interviews to be conducted in IsiZulu rather than English to ensure they fully understood the questions. It is also recognized that merging various cultural groups prevented the researcher from obtaining detailed insights into the practices of any single group.

**Table 1.** Socio-Demographic Profile of Respondents (As Recorded in 2014).

Pseudonym	Age	Ethnic Group	Age When Widowed	Occupation	Reason for Husband’s Death
Vele	37	Ndebele	34	Self employed	Car accident
Iris	42	Swati	34	Administrator	HIV related illness
Thokozile	49	Swati	45	Homemaker	Heart disease
Liffi	62	Pedi	52	Casual worker	Undisclosed long-term illness
Mpho	35	Pedi	31	Home maker	Undisclosed
Nomathemba	52	Xhosa	50	Domestic worker	Burst ulcer (short illness)
Phumzile	39	Zulu	35	Casual worker	Undisclosed short illness
Mahlongwane	43	Zulu	39	Administrator	Cancer
Mrs ngema	52	Swati	50	Home maker	Tuberculosis
Elizabeth	65	Shangaan	65	Cleaner	Natural causes

## Data Collection

Findings in this study relied primarily on data gathered through life story interviews and observations. Field notes were used to compliment and contextualize data gathered through interviews. An interview schedule was created based on the research objectives to collect demographic information, including age, gender, culture, languages spoken, duration of marriage, time since the husband’s death, and the cause of the husband’s death. Women were then asked to share their life stories as widows and on widowhood allowing the researcher to deduce narratives that speak to how they experienced and navigated some practices such the ‘sitting’ and dispossession. The interviews homed in on what transpired after the death of their husbands and solicited a detailed narration of what happened and how they felt and responded to those events. I documented observed behavior or significant emotional responses on my perceptions about each participant, as well as on contextual information about the personal demographics of each participant. In addition to interviews, I had informal conversations with the women that I had easier access to, for example, Vele, Iris, Nomathemba Mrs Ngema, and Phumzile. These conversations were documented in field notes, providing valuable insight into the profound impact of widowhood practices on these women’s lives and enriching the study’s findings. The interviews were taped and transcribed in full, then I followed the standard procedures for qualitative data analysis.

## Ethical Considerations

This study was approved by the university’s ethics committee. The participants were informed about the nature and objectives of the study. Participants were given the essential information about the interview procedure, the expected duration of the interview, they were notified of their free will to withdraw from participating at any point during or after the interview. Each participant signed an informed consent form. Since the topic of discussion was very sensitive and emotional, the participants were informed of the possibility of experiencing emotional distress when recounting their experiences after the loss of a partner. However, none of my research participants experienced that in fact, one of them said “It is comforting to know that there are people out there who care. As I talk to you, I feel you are counselling me”. The recorded interviews were assigned codes and participants’ actual names were avoided during the transcription of findings to uphold confidentiality and anonymity.

## Data Analysis

Braun and Clarke (2022)’s guide to reflexive thematic analysis was used to guide data analysis. Thematic analysis is part of a range of qualitative methodologies that also encompasses narrative analysis (Crossley, 2000; Riessman, 2008) and narrative inquiry (Webster & Mertova, 2007). Thematic narrative analysis employed in this research enables individual units of meaning—words and phrases that convey thoughts, ideas, experiences, and emotions—to surface within the narrative texts. Conducting a narrative thematic analysis allowed for a fair representation of the experiences the women shared during the interviews. The ten interview transcripts provided a rich experience of widowhood and captured complex, detailed descriptions of events that happened during the ‘sitting’.

In the process of analysis, firstly, the researcher read and transcribed interviews to comprehend how widows experienced widowhood rituals. Secondly, the researcher re-read transcripts and selected sections with similar meanings. Thirdly, to add depth and value to their narrative voices, the researcher focused on four interviews and conducted a thorough analysis through prolonged immersion. Fourthly, ideas and themes were noted and coded according to the methods outlined in Braun and Clarke (2022). In the final step, the researcher gained a deeper understanding of the data, by clustering sections with similar meanings. These clusters formed patterns and were described in relation to other meanings. This new and deeper understanding is evident in the descriptions of the participants’ experiences.

## Findings

In this research, I attempted to unpack how widowed women negotiated the culturally sanctioned widowhood practices of the ‘sitting’ and dispossession. I initially anticipated that their accounts would reflect more conventional acts of resistance that include community-based activism, grassroots organizations empowering widows to resist widowhood practices and reclaim agency, and the legal route among others. However, because of the well-documented vulnerability of widows in South Africa (Dube, 2023; Mabunda & Ross, 2023; Manala, 2015; Motsoeneng & Modise, 2020) and how black women occupy vulnerable positions within the family unit in South Africa, I was attuned to the complexities and contradictions of intrafamilial activism and resistance, listening to their narratives against the grain to decipher the kind of resistance that they engaged. The findings indicated that widowhood is a unique site of intersectional oppression, where gender discrimination, economic exclusion, and cultural marginalization intersect. Analyzing widowhood narratives from a feminist framework standpoint has shown how in a patriarchal society such as the one studied here, widowhood positions women as powerless and at the mercy of their in-laws. This often leaves widows socially and economically vulnerable. Widows in this paper not only experienced oppression as women, but also as individuals marked by marital loss, which inevitably led to unique challenges like isolation, silencing, and dispossession. Besides the experience of constrictive nature of the custom of ‘sitting’, widows used it as a space where resistance is forged, and vulnerabilities negotiated. In this space, widows employed a myriad of strategies to challenge the widowhood ritual of dispossession. These included small acts of resistance, such as speaking out, speaking through objects, sending family members to act on their behalf, and disobedience and non-cooperation. Analyzing their narratives, actions, and silences through a feminist lens provided an interpretive framework that revealed these seemingly ordinary acts as politically charged. I begin the discussion of the widowhood practice of the ‘sitting’ as lived, experienced, and articulated by the women and I proceed to discuss the mechanisms they adopted to resist these norms and practices of dispossession.

### Dispossession and the Enforcement of Silence

In women’s narrations of their journey into widowhood, it appears that the custom of ‘sitting’ is usually a site of contestation and confrontation. The women expressed that while ‘sitting’ is a mandatory ritual of widowhood, it is also a point of powerlessness where they are silenced while being dispossessed of their status as wives and of their possessions. Vele narrated her displeasure at having to sit on the mattresses:

After the death of your husband, you must sit on that mattress. You cannot say, no can I sort this out first. You cannot just sit, there is a lot that you must do. Things go very wrong while you are seated and covered with a blanket. You must cover your face while they are busy turning your house upside down. They went through my things. That destroyed my life.

Vele views the practice of ‘sitting’ as an imposed space where she cannot negotiate or even indicate her displeasure. She finds the ‘sitting’ limiting and a space that possibly led to the destruction of her life through the dispossession of her belongings while she was seated. Other participants disclosed that they were ordered to sit because they were perceived weaker than their co-wives and thus easier to dispossess. Iris narrated:

There were two of us married to my husband. After his death, they (in-laws) chose me to sit on the mattress. When they chose you, you are happy because they finally accept that you are his legitimate wife, but now that I think of it, they chose me because they wanted to take my things. They wanted to harass me. They thought ahhh this one, we can do what we want. They didn’t know me. I am a clever woman. I showed them flames shame.

Iris’s narrative illuminates that at times the ‘sitting’ is a space where women are targeted for dispossession, control and domination. She experienced sitting as a practice that invoked feelings of victimization. The targeting of specific women to observe sitting shows that it has become a site of violence, where during their most vulnerable positions, widows are accused of contributing to the death of their husbands, mainly for economic gain. Iris’s story captures this succinctly:

You know I experienced problems from my husband’s side of the family. I was sitting and crying for my husband. You know when a man dies, a woman is to be blamed, and yes, a woman is to be blamed. After that, they won’t like you like for real. I was blamed for the passing away of their child, you see? I don’t know how I can put it across, you know when you are a woman and married and your husband dies, the woman is to be blamed, they say you are the one who killed him forgetting that maybe they saw him when he was sick you see. Even though they know what killed him, but a woman is to be blamed, even though they are very much aware, but a woman is always to be blamed. Therefore, that is how things are (pause). Some of us are used to them. I did not feel sorry because I knew that I was innocent, yes? It was painful to be accused, but I did not want to entertain it because I knew that I am innocent. I knew that time exactly what killed my husband because he was sick, you understand? They said they want all the property; I killed him for this (the house and property)… You see how these things happen?

Iris tells this narrative to the story of her experience of being accused of murder and how this accusation validated the in-laws’ claims to the house and other belongings. Similarly, Liffi detailed her shock at the accusations which she believes were ethnically motivated. She recounted:

My in-laws treated me very badly. You see my child, in-laws treat you like dirt, and I do not have a relationship with them. It was bad; there were talks that “the Johannesburg woman” (Liffi) killed our relative... They accused me of killing him because I wanted his house…

After her husband’s death, Mpho somberly narrated her ordeal:

It was difficult, shame. There was a lot of tension between my family and his family. His family was saying I should hand over the house and the cars. They were saying I should sell the house and the cars so that we share everything. According to them, no one was supposed to win. When I was seated on that mattress, they accused me of killing my husband, they said I knew exactly what killed him and I killed him because I wanted to benefit from his death and take all his wealth, therefore everything was supposed to be sold so that I do not benefit from killing him.

Such utterances were invariably common to the conversations I had with the widows regardless of whether they married in civil marriages or customary marriages or if their husbands died intestate or not. The above narratives highlight that widowhood in a culturally cohesive society such as South Africa is a challenging phase. Central to the narratives of women was that during the initial phase of mourning that starts with the ‘sitting’, women are accused of killing their husbands as a precursor to widow dispossession. By accusing them of murder, their in-laws assume the authority to exclude the widow from the family and redefine her status within the family as that of an outcast and enemy. This allows them to dispossess the widow of property and their status as a family member.

Silence is an integral rite while observing the custom of the ‘sitting’. It is one of the means through which widow dispossession is enforced. While the widow mandatorily sits, the primary expectation is to show respect to the dead and his family by observing silence. Iris said:

I knew that I was supposed to sit and cover myself with a blanket… The thing is that I was not ready. You know, you are never ready for these things. … Suddenly, there were people everywhere in my house, telling me what to do. Imagine even dressing me up… I tried to tell them that I did not want to do some things but my mother and some of her church people said I must allow them to lead the process, and I must not be seen talking, people will think that I am happy that my husband is dead… I did not want people to think that way. I was sad and confused, you know when you watch it happen to other people, you do not feel it... you do not know how it feels. You see, I am used to doing things by myself, arranging things, but to stay silent was the worst for me. Especially when people were busy sharing my things amongst themselves. My husband’s brothers, sisters and mother started identifying things that they wanted, and I was expected to just sit, watch and cry… It was a mess; I can tell you!

The imposition of silence in this regard ensures the smooth dispossession of property as per contemporary practices of culture, the widow could not resist or refuse. For instance, Liffi lamented bitterly on her incapacitation during the rite of the ‘sitting’:

My in-laws treated me very badly. You see my child, in-laws treat you like dirt, what made it worse is that I couldn’t even say anything. When you are there, you show respect. You don’t argue or raise your voice. Even looking people in the face is not allowed. I just looked down… yooo the pain was big. I have never felt that much pain. I don’t think I will ever be myself again… and I do not have a relationship with them. It was bad…

These conversations reveal that the primary requirement for the widow when observing the rite of the ‘sitting’ is silence and this incapacitates them. Vele further added a critical aspect of the experience of silencing:

“I felt that like I was suffocating, everything was closing in... I couldn’t breathe. I could hear people talking, accusing me of killing my husband, who, mind you, died from a car accident…. They were making decisions about my life and my children…. I knew right there that I was going to be a different widow. A widow who does not take nonsense…

While Liffi accepted the recommended silence and “just looked down”, Vele was willing to push the boundaries to protect her assets. She wanted to be a “different widow,” by breaking the boundaries that were imposed on her. The feeling of suffocation was motivation for her to break free.

The above excerpts highlight how the predominant requirement of the ‘sitting’ – silence, left them feeling violated, suffocated and powerless. During this process, and through practices of violation and dispossession, these women were transposed from their statuses as wives in the family to those of an outsider. As such, this validated claims for dispossession. While in their moments of powerlessness and hopelessness, these women engaged in self-conscious acts of activism. Accordingly, the frictions that emanated from this contested space of liminality introduce the circumstances that lead to the emergence of widowhood activism.

### Multiple Acts of Resistance

While Sossou (2002) suggested that widows in Africa are silent victims with little or no political agency, my research contradicts this position. Indeed, what emerges are resilient, active, and resourceful widows who can draw on very limited resources to negotiate and resist widowhood dispossession. Common in many of the women’s narratives were descriptions of how their activist identities were forged through low-level confrontations and small acts of resistance to especially the male in-laws and the husband’s side of the family more generally who assume a superior position by being aligned to the male side of the family.

#### Amplifying Voices: Speaking Out

The narratives highlighted the lived experiences of breaking free from the socio-cultural expectation of observing silence and widow dispossession during the ‘sitting’. Women’s voices underline that they couldn’t sit and observe while they felt vulnerable and victimized. Iris narrated how she disobeyed this custom by talking and arguing during the ‘sitting’, while people kept telling her to keep quiet: “People told me to keep quiet; I would speak my heart out when I was sitting on that mattress….” Iris decided to speak against normative accusations that she killed her husband. She explained that his family knew what had killed him and about his disease. Therefore, she “did not want to entertain it” and “did not feel sorry”. By speaking out against such accusations, she claims that she crippled their efforts at dispossessing her of her property. She reflected nine years after the initial interview:

I told them (the in-laws) that they knew that their son did not even have a good job. He was dependent on me. How can they say they are taking property? That was ridiculous. I asked them, where they think he got the money to buy a house from. Like people decide to be stupid… I could not be quiet and allow people to steal my house, my car, my property… I told them that he was lucky to have married me in the first place. I mean look at me... I do not have a relationship with any of them now.

In 2014, Iris had said “They should know that I work, and this is my money. Their son had no car, even a driver’s license, he could not even drive.” This reflects on Iris using her voice to disrupt a system that is designed to dispossess and silence her. By suggesting she worked hard to acquire their property, contributing more than her husband, who did not have a good job, Iris challenged the inherent bias in a patriarchal system that places men as more economically viable than women. Iris’s comments allude to how resistance played out under constrained circumstances. She had the agency to choose between conforming to the prescripts of widowhood - silence or voicing her concerns and she chose the latter.

When confronted with the possibility of losing her property and other belongings, Mpho decided to forego the ritual of silence:

I knew that if I keep quiet, I will lose. I asked for my brother-in-law to come into the room to discuss issues and he refused and said he won’t talk to me and has nothing to say to me... that the family has decided, and their word is final. That’s when I went crazy, yoooo I went crazy. I was like I am not going to allow this. I started shouting, talking very loudly, it was like all the hurt came back…. I felt so much pain. I just talked, I was crying and talking. People heard the noise; I was held down. I was trying to stand up… It was crazy, I didn’t care anymore…

Mpho’s statement reveals an incisive blending of vulnerability and agency which informs the type of resistance she engaged, which was shouting and talking to attract the attention of her in-laws and other bystanders. This, she hoped, would result in a positive outcome for her. In addition, her words show her choice to emphasize her vulnerability, which is influenced by her position during the rite of the ‘sitting’, a site where silence and vulnerability are lived. Through refusing imposed silence, widows defied the custom of silence by speaking, sometimes shouting to change the circumstances that were being designed for them. This gave them control over their properties and over their lives. This indicates that widows (continue)

#### Speaking Through Objects

In other instances where silence is commanded, objects are then brought in to speak on their behalf. Having failed to convince her in-laws through shouting and speaking out, Mpho engaged another tool which she thought was more powerful than words. Mpho decided to give agency to the most powerful object that she had - the marriage certificate. Together, with other women who were married in a civil union, the marriage certificate usually referred to as “the paper” became a powerful tool of resistance. After speaking out, Mpho’s in-laws insisted that she was not a legitimate wife as she was not formally married to her husband:

They then said I was not married; I told them that we were married; I have the paper so they cannot sell or distribute those things without my consent. That angered my in-laws, his brother lost it, and he slapped me very hard across the face. It was difficult for me. My brothers and uncles were angry, and they started fighting with his family. There was a lot of chaos. They told me that I could not say I am married, there was no ceremony to accept me into the family therefore, they do not recognize the marriage, and I am just like all those other women he had children with. What surprised me is that lobola (bride price) was paid but we did not have a white (formal) wedding. My husband and I went to home affairs and registered the marriage.

Similar instances of the marriage certificate being invoked as an object that has the potential to vindicate them from dispossession and other related operations. For instance, Iris explained:

No, we did not share any property. Isn’t I being the one remaining behind and I had the paper (marriage certificate)? I mean the marriage certificate conquers all. So, they did not have power because I had the marriage paper.

These narratives highlight that in the absence of speech or when speech does not work, physical objects are used to communicate. They relied on the marriage certificate as a legal document that grants them rights to their property as well as a position within the family. It was used as ‘symbolic capital’ to challenge cultural demands (cf. Bourdieu [1998] 2001). Consistent with Bourdieu’s theorization of the concept of symbolic capital, women used the marriage certificate as a recognized symbol of a legally and socially sanctioned union to claim not only for recognition as legal wives of the deceased but some form of power. Even in instances where its cultural legitimacy was questioned, the fact that it is a legally binding document challenges the orthodox customary laws and practices. However, it was not available to women who were in customary marriages. Leaving them to tap into other resistance sources that were available to them.

#### Actions Through Proxy

When direct speech or action was too risky, ineffective, and inappropriate some women resorted to silence to communicate dissent and resistance. Thokozile had to be creative after realizing that she stood to lose significantly. She took advantage of the restrictions and sacredness of the rite of the ‘sitting’ to hide some of her personal valuables:

When I heard that they wanted to take my things. I had to act fast. I called my brother and asked him to ask for the keys to the van from my brother-in-law, he was already using my husband’s car. I told my brother to ask nicely and say he had to go buy groceries…. I didn’t think they will give him, but when they gave him, I was like phewww thank God. I took the keys and sat on them, under me there already were policy documents, his identity documents, life insurance, work papers, everything. I sat on everything. You see when you are there, you don’t stand up. When I wanted to go to the toilet, I asked my sister to sit there…. People did not know what we were up to (laughs)…

For Thokozile, silence was a conscious decision to exert control over her situation and protect herself and her interests. This strategic form of resistance that included more than just sitting on objects was employed. Thokozile and the other women in this study used trusted relatives as their eyes, ears, and mouths. As such, while they were silent, they had people to act and speak on their behalf. Thokozile’s siblings and children had to stand on her behalf while she sat and observed tradition:

Because my sister had gone through this, she quickly came to be my side… She fought with people for me… she updated me on everything… she would ask me how I wanted things to be done and did things for me. My daughter was a bit grown, and she would tell people that my mama won’t like this and that. My brother too, I was updated on everything. I knew what people wanted to do and what they were thinking.

Thokozile’s actions were not just silence, but actions through proxy. She extended her agency beyond herself to people around them to act on their behalf. Although women’s social positions did not allow them to organize high-profile demonstrations, they either stood alone or stood with a few other women, the mother, sisters, and male sympathizers such as male children and brothers. Kanyoro (2001, p. 51) found that in instances where women do not have the resources to fight, people must recognize of women’s “choked silence” as a form of resistance. This study recognizes women’s ability to extend agency beyond themselves by listening to their choked silences. The act of instructing family and friends, for example, to do certain things for them or the use of objects to fight for them enabled the widows to perform certain acts of resistance while seated. Their bodies extend, they outsource eyes, ears, feet, hands, and intelligence through the networks around them.

#### Disobedience and Non-Cooperation

Some women expanded these proxy actions to encompass acts of disobedience and non-cooperation. Vele’s narrative highlights her deliberately ignoring certain rules, refusing to follow orders, and actively resisting directives as a form of protest or defiance:

I did not follow their instructions, some of the things were ridiculous to me. Imagine they wanted me to sit and cry while they were damaging my children’s future and making funny decisions about my life. … They also said I must sit down, keep quiet and not do anything. I never listened to all that. The next day after he died, I drove 127 km to the mortuary where they had put his body. They said I must not drive, so who was supposed to drive my car? The people who said I killed him. No, I knew I was alone and before my parents and brother came, I drove myself around signing papers (policy documents) and arranging the funeral. You can’t allow people who want to hurt you to do things for you…. I did things on my own... I was going to lose everything if I just sat and cried.

Vele refers to a refusal to take instructions as her preferred response to the possibility of dispossession. By driving around and signing policy documents, she arrested efforts to rob her of her inheritance. In the above excerpts, Thokozile and Vele demonstrated how they employed acts of non-cooperation and disobedience to defy certain customs including that of dispossession. These forms of resistance can be powerful and effective, demonstrating dissent and resistance and amplifying concerns and demands for those who are silenced.

## Discussion

In this work, I was particularly interested in understanding the resistance tactics of women who had gone through widowhood rites and practices in negotiating widowhood dispossession. As such, this study found that women engaged in ‘small acts of resistance’ (Scott, 1985), which took the form of subtle actions that challenged the patriarchal power structures. Their acts included behaviors such as non-compliance, quiet defiance, and other forms of passive resistance that, while not overtly confrontational, serve to assert agency and resist the oppressive nature of the custom of the ‘sitting’ and its relational consequence of widow dispossession. Women’s continued opposition can be seen as a ‘prosaic’ struggle (Scott, 1985, p. 6) but daring because their positions as *makoti* (daughter in law) does not allow for any activity that exceeds socio – cultural prescriptions of how a *makoti* should behave. In as much as in some instances their resistance was overt, especially in terms of speaking out, most of it was covert and less self-consciously activist, more inclined to ‘foot-dragging and evasion’ to which Scott refers (Scott, 1985, p. 8). The fewer instances where these women resorted to overt forms of resistance were in response to the gravity of the circumstances in which they found themselves in.

The narratives above illustrate how widowhood serves as a distinct area of intersectional oppression where structural inequalities are embedded in social institutions like the family. However, instead of adhering to the oppressive norms presented to them, widows engage in different resistance strategies. These highlight the new type of widow (an activist) who engages unusual practices of resistance while revealing how widows use these strategies as a form negotiating an oppressive system, where overt demonstrations or resistance would be harmful to them. This demonstrates how mundane activities can be examined as a form of resistance and that while they are typically regarded as too minor to qualify as activism, they foster meaningful change in the lives of women. Their narratives highlight the vulnerabilities experienced by the widows as well as the potential for transformation (Akinduyo & Theron, 2024). Resisting while seated is the default response for these women. It highlights the rigidity of some cultural norms and their constrictive nature for women who find themselves having to conform to them.

The rite of the ‘sitting’ is not just a symbolic act of mourning, but a political one where the widow is silenced, dispossessed, marginalized and rendered powerless. Kotzé et al. (2012), p. 754) reported similar findings regarding the practice of the ‘sitting’ and other customs, showing that widows can adopt various agentic roles by accepting, questioning, resisting, and/or transforming cultural mourning practices. However, in their findings, they argue that women adopt these stances when it is easier or feasible for them. For this study, I find that it is when women have no alternative that they resort to resisting in subtle ways. These widows’ narratives indicate that they resort to resistance when they are at their most vulnerable. In this context, the ‘sitting’ is a symbolic act of loss where loss is enacted and embodied through a myriad of rituals and loss is not just losing the husband, rather it is the loss of voice and property. It is also a site where women resist its current forms as a custom that serves the interests of patriarchal practices of widow dispossession. Widowhood, through its customs like the ‘sitting’ is a liminal phase where the widow is turned into both a subject and object of mourning rituals (Ramphele, 1996). While the ‘sitting’ is a position where the widow embodies of loss and suffering, I highlight how they use the limited resources at their disposal to push against collateral customs like widow dispossession.

Undoubtedly widowhood is gendered, as the ‘sitting’ is observed by women and dispossession mostly happens to women. While being aware of the repercussions, sometimes women their own insist on observing mourning rituals like the ‘sitting’ as a way of honoring their departed spouses, this can be read as a refusal to be indifferent to loss (Kotzé et al., 2012). However, they adopt different positions of agency in response to oppressive practices, remaining dedicated to a “sense of human vulnerability and shared responsibility” (Butler, 2004, p. 30), and acknowledging their connection to others (Derrida, Brault, & Naas, 1996).

The findings of this study indicate that widowhood activism comes at a personal cost. Widowhood is a social construct that is created through control and restriction. In this way, countering it upsets the domination and creates friction between the widows and those who benefit from patriarchal power structures. As such, resisting dispossession or disrespecting the prescripts of the ‘sitting’ was met with either physical violence, a disruption in family relations and eventual isolation. Gqola (2021) emphasizes that dismantling patriarchy is both necessary and risky. It often comes with significant personal, social and economic repercussions.

## Conclusion

This paper captures the resistance strategies that widows engage in resisting the widowhood custom of dispossession. Their narratives debunk the common stereotype (in literature and society) that they are silent victims of widowhood rituals. From their positionalities as seated, silenced, and dominated, widows in this article resist the ritual of dispossession through engaging in subtle acts of resistance. These include speaking out, using objects to lay claims to their properties, engaging family members to fight on their behalf and being disobedient and non-cooperative. These actions in a way shape their identities as often unrecognized and unacknowledged activists operating on the peripheries of society. Understanding their identities as activists involves reading or listening closely to their narratives and looking for those hidden acts that have the potential to bring some positive change in women’s lives. The argument put forward is that the widowhood practice of ‘sitting’ creates a site within which enforced silences and widow dispossession are ritualized and where widowhood activism is born. At its core, the paper first argues that while the custom of the ‘sitting’ is a long-standing tradition of mourning, it has become a political space that enforces silence and domination and second, while observing enforced silences and the physical act of sitting, widows take up the invisible act of standing against dispossession. As such, I read the practice of the ‘sitting’ as more than just a rite of passage for widowhood, rather as an instrument of control and dispossession and a space where widowhood activism emerges. Widowhood activism raises important questions about pain and suffering as central to widowhood, and the extent to which widows become both subjects, objects, and victims of mourning. Understanding the personal accounts of widows and their engagement with certain cultural practices from a feminist standpoint offers us a framework for theorizing how their activism and resistance can foster broader social change.

## Limitation and Future Research

While this study provides valuable insights into widowhood resistance strategies to dispossession, several limitations should be noted. First, the study focused on two cultural practices, that of the ‘sitting’ and of dispossession in an urban setting. A broader take on the different practices, involving a broader sample of widows would be beneficial to understand the complexity of resistance especially in poorer and more rural contexts. Second, participants in this research came from a wide range of ages, ethnicities and varying economic classes, while this was an advantage in this research in terms of getting a sense of the commonalities in experience and responses to widowhood, a more focused approach could be conducted to ascertain how these different dynamics influence their chosen resistance strategies. These limitations point to valuable areas for further exploration. Future research could delve into the broader cultural experiences of widowhood and activism, with attention to the diversity among Black African women, who navigate varying societal structures and forms of domination. Recognizing this diversity can deepen understanding of widowhood within broader social and cultural contexts.

## Notes

1. Examples include The Recognition of Customary Marriages Act (1998). This act ensures that customary marriages provide equal property rights to spouses, granting women legal recognition and protection in marriage and property matters. The Reform of Customary Law of Succession and Regulation of Related Matters Act (2009). This reform prioritizes gender equality in inheritance rights, ensuring that women, including widows, have rightful claims to family property under customary law.

2. I use widow and woman interchangeably in this paper to avoid reproducing the loss of identity that women go through in widowhood. I hope in some way to retain their identity as women first and foremost before they are abruptly stripped of their identity.

3. I classified respondents as poor to lower middle class based on a combination of indicators, including income levels, employment status, access to essential resources, and general economic stability. According to Stats SA (Living Conditions Survey, 2014/2015) the average monthly salary in South Africa’s formal sector, often used as an indicator of the middle class, stood between R1500 (150 US dollars) to 10 000 South African Rand, (1000 US dollars) at the time. Except for Iris and Vele, most of the participants were either working as domestic workers, some were into small informal trading (e.g., selling tomatoes at the sides of the roads) and a few were earning were working in the lower paying formal jobs as office administration and office cleaning and their husbands were mostly in blue collar employment as general workers or manual laborers. These, even with a combined salary would barely make it to 1000 US dollars at the time.
